# Case Report of Thromboembolism Prophylaxis in a Burn Patient With COVID-19

**DOI:** 10.7759/cureus.36009

**Published:** 2023-03-11

**Authors:** Mohammed Alfawzan, Asma Alhabib, Eman Alshammari, Muhammad M Ulhaq, Ahmed Eldali, Rawan M Alhazmi, Dana K Alsarhani

**Affiliations:** 1 Plastic and Reconstructive Surgery, King Abdulaziz Medical City, Riyadh, SAU; 2 Plastic Surgery, National Guard Health Affairs, King Abdulaziz Medical City, Riyadh, SAU; 3 Plastic Surgery, King Faisal Specialist Hospital and Research Centre, Riyadh, SAU; 4 Plastic Surgery, King Abdulaziz Medical City, Riyadh, SAU; 5 Ophthalmology, King Saud Bin Abdulaziz University for Health Sciences College of Medicine, Riyadh, SAU

**Keywords:** covid-19, thromboprophylaxis, deep vein thrombosis, pulmonary embolism, venous thromboembolism

## Abstract

The recent coronavirus disease 2019 (COVID-19) pandemic has worse medical consequences, especially when affecting people with comorbidities such as diabetes, lung disease, hypertension, burn and trauma. The pathophysiology of COVID-19 infection includes thromboembolic events that were described in previous studies as a risk of venous thromboembolism (VTE). This risk is higher in burn patients, especially in the electrical type, which is generally attributed to their hypercoagulable state.

This article reviews a detailed history, examination, and investigations of a 38-year-old male hospitalized burn patient with COVID-19 infection. Although on chemical thromboembolic prophylaxis, the patient developed extensive pulmonary embolism (PE) and, more interestingly, had atypical PE signs and symptoms. The present case aims to develop a special venous thromboembolism prophylaxis protocol between prophylactic and therapeutic dosages for COVID-19 burn patients.

## Introduction

Patients with coronavirus disease 2019 (COVID-19) present with a variety of clinical spectrum, ranging from asymptomatic, mild respiratory or gastrointestinal symptoms to severe pneumonia and multiorgan failure [1]. Damage to the lung epithelium and an immune reaction against the virus are both characteristics of COVID-19's pathogenesis. The inflammatory cascade, which is followed by acute respiratory disease syndrome (ARDS) and multiorgan failure, is the leading cause of death by COVID-19 [1,2].

Researchers indicated that excess inflammatory process predisposes thrombotic events, most commonly pulmonary embolism (PE) [2]. Venous thromboembolism (VTE) in COVID-19 patients leads to disease-induced coagulopathy in addition to respiratory distress or acute infection [3]. A multicenter prospective cohort study revealed that despite anticoagulation, a high number of patients with ARDS secondary to COVID-19 had a five-fold risk of PE compared to matched non-COVID-19 (11.7% vs. 2.1%), among which 2.7% developed hemorrhagic complications. Hence, a higher anticoagulation target than in usual critically ill patients should, therefore, probably be suggested [4]. A study from the Netherlands demonstrated that despite routine standard thromboprophylaxis, 20% of COVID-19 patients developed VTE (26%, 47%, and 59% in the ICU, 5.8%, 9.2%, and 9.2% in the ward setting) at seven, 14, and 21 days [5].

In a previous study, local protocol for thromboprophylaxis in participating centres for patients admitted to the intensive care unit recommends against the use of a therapeutic dose of oral anticoagulants for VTE prophylaxis or prevention of COVID-19 progression [6]. On the other hand, some authors and medical societies advised using intermediate or therapeutic dose anticoagulants for VTE prophylaxis [4,7].

Burn trauma itself indicates an acquired hypercoagulable state, coexisting with COVID-19 infection will increase the risk of thrombosis. This occurs because all three aspects of Virchow's triad-stasis, endothelial injury, and alterations in blood components will be more prevalent [8,9]. Of all types of burns, the electrical burn was found to be the most independent risk factor for developing VTE [9]. Consequences of VTE in prophylactically anticoagulated burn patients were frequently reported in the medical literature [10]. Considering that the standard dose of anticoagulant may be insufficient, the dose should be adjusted based on anti-factor Xa level or applying specific dosing formulas that include patient weight, and total body surface area (TBSA) is recommended [8].

There is no standard approach among burn centers either to do VTE prophylaxis in burn patients or not. Moreover, the VTE guidelines have no section specific to burn patients due to limited research in the field of burn DVT prophylaxis [8]. A study from the United States found that two-thirds of the burn centers were using thromboprophylaxis in their burn population [10]. Debate exists regarding the effective thromboprophylaxis plan for hospitalized COVID-19 patients. The present case aimed to highlight the importance of having an effective venous thromboembolism prophylaxis protocol between prophylactic and therapeutic dosages for COVID-19 burn patients that will ultimately affect VTE incidents and improve quality of life.

## Case presentation

On February 5, 2022, a 38-year-old male, who had no past medical history (PMH), was brought to the Burn Center of Abdul-Aziz Medical City for further evaluation of his electrical burn injuries. The patient was found to have 33% TBSA burns in his back, trunk, lower extremities, and upper extremities, but his lung exam was unremarkable. Following the trauma protocol, computed tomography (CT) was performed for chest CT, abdomen, and pelvic CT, in addition to ECG, and all were unremarkable.

On arrival, the patient was positive for COVID-19 on admission, and he reported having three days of sore throat, malaise, and fever, which started one week before the admission. As per COVID-19 interim guidelines, he was isolated for 10 days on a prophylactic dose of heparin 5000 subcutaneously three times a day.

While in the hospital, he experienced several spiked fever episodes. A septic work-up revealed positive urine and wound cultures. The infectious disease team was notified and prescribed him intravenous β-lactam antibiotic (meropenem) for 14 days starting from February 19.

On February 20, the patient was transferred to the operation room for debridement of second- and third-degree burns and skin graft in one session for the anterior trunk and left lower limb. One unit of blood was given intraoperatively.

**Figure 1:**
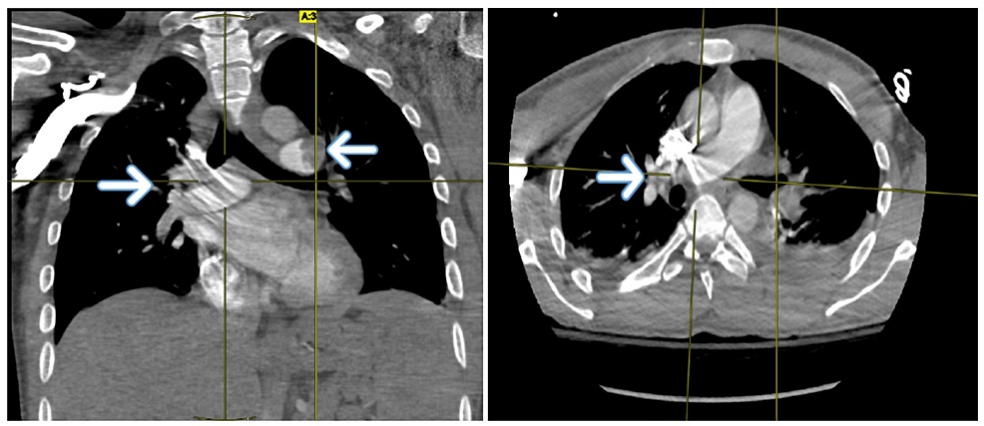
Chest angiography showing bilateral extensive central and peripheral pulmonary embolism as described above (white arrows)

Four days later, the patient complained of localized chest pain associated with spikes of fever up to 38.9, episodes of borderline hypotension 91/63, and tachycardia. He was hypoxic up to 88% on two liters of nasal cannula oxygen. Further investigation of chest angiography (Figure 1) revealed bilateral extensive central and peripheral pulmonary embolism with findings of right lower lobe hypodense focal consolidation, which was suspicious for pulmonary infarction.

After discussing the case with the internal medicine team, they decided to provide a heparin infusion. Following that, the patient’s condition improved gradually, and his vital signs started to be stabilized. Lastly, he was planned to be discharged on anticoagulant medication (apixaban) for three months and afterward to be followed by a coagulation clinic.

## Discussion

Due to the hypercoagulable state resulting from disturbances in the Virchow's triad, thrombosis of the capillaries and small caliber vessels and rarely medium and large caliber vessels was associated with severe burns [8,9]. Among all burn types, electrical burns were encountered to be one significant risk factor for venous thromboembolism [9]. Correspondingly, COVID-19 is a hypercoagulable state which may also predispose to both venous and arterial thrombo-embolic events due to inflammation of cytokines, hypoxia, immobilization, and diffuse intravascular coagulation [1,2]. The present case emphasizes the risk of venous thromboembolic complications in patients diagnosed with burns and the need to remain alert to this possibility.

Furthermore, numerous studies have been conducted to investigate the frequency of PE among COVID-19 patients, and their findings showed that the incidence of PE is higher in COVID-19 patients compared to non-COVID-19 patients [4,5]. In the presented case, the patient was at a high risk of VTE. Thus, he was started on prophylactic anticoagulants; however, 14 days later, he was diagnosed with PE. This finding is similar to a previous prospective cohort study that showed a higher prevalence of thrombotic complications in COVID-19 patients (11.7%) compared to non-COVID-19 patients (2.1%), and these thrombotic events occurred despite prophylactic or therapeutic anticoagulation [4]. Similarly, a single-center cohort documented that among the patients who were hospitalized with COVID-19, 38% were admitted to the intensive care unit (ICU). During a median follow-up of seven days, 20% of patients were diagnosed with VTE, of whom 13% had symptomatic VTE, despite routine thrombosis prophylaxis [11].

On the other hand, one study found that in-hospital mortality for patients being treated with anticoagulant medication was 22.5%, with a median survival of 21 days, as opposed to 22.8% and median survival of 14 days in those not receiving treatment-dose anticoagulant. Also, it was found that patients with treatment-dose anticoagulant medication were more likely to require invasive mechanical ventilation (29.8% vs. 8.1%; p < 0.001) compared to patients who received prophylactic doses or did not receive anticoagulant [10]. Analogously, there is an opposing recommendation from the COVID-19 treatment guidelines panel to the use of a therapeutic dose of oral anticoagulants for VTE prophylaxis or progression of COVID-19 [6].

## Conclusions

The incidence of venous thromboembolic disease due to COVID-19, despite the use of preventive and therapeutic dosage, formulated the hypothesis of unique pathophysiology. The present case supported the idea of encountering coagulopathy complications in any case with SARS-CoV-2 infection, especially in patients with burns, by standardizing the dosing higher than the routine prophylactic dose.
